# Regional Clinical and Biochemical Differences among Patients with Primary Hyperparathyroidism

**DOI:** 10.4274/balkanmedj.2015.0865

**Published:** 2017-01-05

**Authors:** Özer Makay, Beyza Özçınar, Turgay Şimşek, Cumhur Arıcı, Bülent Güngör, Serdar Özbaş, Tamer Akça, Ali Uğur Emre, Güldeniz Karadeniz Çakmak, Müfide Akçay, Bülent Ünal, Mustafa Girgin, Sadullah Girgin, Semih Görgülü, Atakan Sezer, Adem Karataş, İbrahim Ali Özemir, Nihat Aksakal, Serap Erel, M. Ümit Uğurlu, Ali İlker Filiz, Can Atalay, Ali Uzunköy, Uğur Deveci, Çetin Kotan, Gökhan İçöz, Yavuz Kurt, Abut Kebudi, N. Zafer Cantürk, Yeşim Erbil, Rumen Pandev, Bahadır M. Güllüoğlu

**Affiliations:** 1 Department of General Surgery, Division of Endocrine Surgery, Ege University School of Medicine, İzmir, Turkey; 2 Department of General Surgery, Division of Endocrine Surgery, İstanbul University İstanbul School of Medicine, İstanbul, Turkey; 3 Department of General Surgery, Kocaeli University School of Medicine, Kocaeli, Turkey; 4 Department of General Surgery, Division of Endocrine Surgery, Akdeniz University School of Medicine, Antalya, Turkey; 5 Department of General Surgery, Ondokuz Mayıs University School of Medicine, Samsun, Turkey; 6 Department of General Surgery, Güven Hospital, Ankara, Turkey; 7 Department of General Surgery, Division of Endocrine Surgery, Mersin University School of Medicine, Mersin, Turkey; 8 Department of General Surgery, Bülent Ecevit University School of Medicine, Zonguldak, Turkey; 9 Department of General Surgery, Atatürk University School of Medicine, Erzurum, Turkey; 10 Department of General Surgery, İnönü University School of Medicine, Malatya, Turkey; 11 Department of General Surgery, Fırat University School of Medicine, Elazığ, Turkey; 12 Department of General Surgery, Dicle University School of Medicine, Diyarbakır, Turkey; 13 Department of General Surgery, Gülhane Training and Research Hospital, Ankara, Turkey; 14 Department of General Surgery, Trakya University School of Medicine, Edirne, Turkey; 15 Department of General Surgery, İstanbul University Cerrahpaşa School of Medicine, İstanbul, Turkey; 16 Department of General Surgery, İstanbul Medeniyet University School of Medicine, İstanbul, Turkey; 17 Department of General Surgery, Ankara Training and Research Hospital, Ankara, Turkey; 18 Department of General Surgery, Marmara University School of Medicine, İstanbul, Turkey; 19 Department of General Surgery, Okan University School of Medicine, Istanbul, Turkey; 20 Department of General Surgery, Ankara Oncology Training Hospital, Ankara, Turkey; 21 Department of General Surgery, Harran University School of Medicine, Şanlıurfa, Turkey; 22 Department of Gernral Surgery, Sultan Abdülhamid Training and Research Hospital, İstanbul, Turkey; 23 Department of General Surgery, Yüzüncü Yıl University School of Medicine, Van, Turkey; 24 Department of General Surgery, Division of Endocrine Surgery, Tsaritsa Yoanna University School of Medicine, Sofia, Bulgaria

**Keywords:** hyperparathyroidism, clinocopathological features, regional differences, Turkey, Bulgaria

## Abstract

**Background:**

Environmental habitat may play a role in clinical disparities of primary hyperparathyroidism (pHPT) patients.

**Aims:**

To compare preoperative clinical symptoms and associated conditions and surgical findings in patients with pHPT, living in different geographical regions from the Black Sea, Mediterranean and Anatolia regions.

**Study Design:**

Retrospective, clinical-based multi-centric study of 694 patients with pHPT.

**Methods:**

Patients from 23 centers and 8 different geographical regions were included. Data related to baseline demographics, clinical, pathologic and treatment characteristics of 8 regions were collected and included age, gender, residential data, symptoms, history of fracture, existence of brown tumor, serum total Ca and p levels, serum parathormone (PTH) levels, serum 25-OH vitamin D levels, bone mineral density, size of the resected abnormal parathyroid gland(s), histology, as well as the presence of ectopia, presence of dual adenoma, and multiple endocrine neoplasia (MEN)- or familial-related disease.

**Results:**

The median age was 54. Asymptomatic patient rate was 25%. The median PTH level was 232 pg/mL and serum total Ca was 11.4 mg/dL. Eighty-seven percent of patients had an adenoma and 90% of these had a single adenoma. Hyperplasia was detected in 79 patients and cancer in 9 patients. The median adenoma size was 16 mm. Significant parameters differing between regions were preoperative symptoms, serum Ca and p levels, and adenoma size. All patients from South-East Anatolia were symptomatic, while the lowest p values were reported from East Anatolia and the largest adenoma size, as well as highest Ca levels, were from Bulgaria.

**Conclusion:**

Habitat conditions vary between geographical regions. This affects the clinicopathological features of patients with pHPT.

Adenomas are the leading cause of primary hyperparathyroidism (pHPT). Throughout the last decades, decreasing size of the adenoma seems to challenge the preoperative work-up and intraoperative management. There are even reports mentioning significant differences between patients from different regions (1), geographic variation between pHPT patients has not been well defined throughout the literature. Although some Western and Eastern studies report large size and/or very high parathyroid hormone levels being involved with increased risk of malignancy (2,3), this has not been supported by others (4). However, nonclinical factors like the environmental habitat may play a role in the disparities between pHPT patients between regions. To the best of our knowledge, the direct impact of geography on clinical and biochemical data have been neither investigated, nor addressed. Thus, here we aimed to compare the preoperative clinical symptoms and associated conditions and surgical findings in patients with pHPT, living in different geographical regions of the Black Sea, Mediterranean and Anatolia regions in Turkey as well Bulgaria as a whole.

## MATERIALS AND METHODS

**Figure 1:**
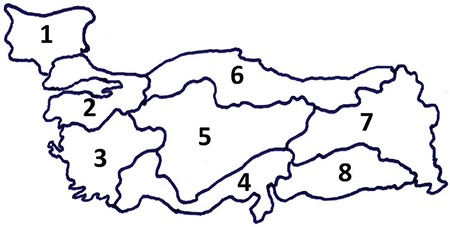
Geographic boundaries of the 8 study regions. ^1^Bulgaria, ^2^Marmara, ^3^Turkish Aegean, ^4^Turkish Mediterranean, ^5^Central Turkey, ^6^Turkish Black Sea, ^7^Eastern Turkey and ^8^South-East Turkey

This is a retrospective study of patients with pHPT treated with parathyroidectomy in 23 centers across Turkey and Bulgaria. Data were collected on all consecutive patients using a standardized template that was finalized and distributed to all centers. The use of these data was consistent with the regulations of the Institutional Review Board of Ege University (Approval number: 16-8.1/36) and received approval. Members of each center performed the collection separately for patients, who had lived for more than 10 years in one of the geographical regions defined below, operated on between January 2010 and September 2012. To account for any regional differences in practice patterns and outcomes, the data were stratified to account for 8 different regions: ^1^Bulgaria, ^2^Marmara, ^3^Turkish Aegean, ^4^Turkish Mediterranean, ^5^Central Turkey, ^6^Turkish Black Sea, ^7^Eastern Turkey and ^8^South-East Turkey (Figure 1). Baseline demographic, clinical, pathologic and treatment characteristics of the 8 regions were collected and included age, gender, residential data, symptoms, history of fracture, existence of brown tumor, serum total Ca and p levels, serum parathormone (PTH) levels, serum 25-OH vitamin D levels, bone mineral density, size of the resected abnormal parathyroid gland(s) (largest when more than one gland was removed), histology, as well as presence of ectopia, presence of dual adenoma, and MEN- or familial-related disease. Preoperative work-up with ultrasound, scintigraphy, computerized tomography (CT), magnetic resonance imaging (MRI), as well as type of surgery, type of anesthesia and complications, have been questioned.

The diagnosis of pHPT was based on the presence of elevated calcium and PTH levels in the absence of chronic kidney disease. Diagnosis of pHPT was carried out by specialists. Vitamin D deficiency was defined as a serum 25-hydroxy-vitamin D level below 50 nmol/L. Patients underwent localization tests and on the basis of the preoperative work-up, parathyroid surgery was performed. Persistence of the disease was defined as persistence of hypercalcemia immediately after surgery, while recurrence was the presence of hypercalcemia developing after 6 months of surgery in a post-surgery normocalcemic patient. Asymptomatic pHPT cases were selected for surgery according to the 2009 guidelines for the management of asymptomatic pHPT of the third international workshop [Bilezikian, Khan, Potts; Third International Workshop on the Management of Asymptomatic Primary Hyperthyroidism. Guidelines for the management of asymptomatic pHPT: summary statement from the third international workshop. J Clin Endocrinol Metab 2009;94:335-9].

The histological criteria for parathyroid carcinoma used were the presence of parenchymal mitoses, trabeculated parenchyma including a thick fibrous band and capsular or vascular invasion. Capsular or lymphovascular invasion were the features accepted as the most specific markers of the diagnosis of parathyroid carcinoma.

Statistical analysis was carried out using Statistical Package for the Social Science release 22.0 for Windows software package (SPSS; IBM Inc., Chicago, USA). Regional outcomes were assessed. Differences between the various clinical, pathological and treatment variables across the 8 separate regions were analyzed using The Mann-Whitney U test and Student t test for the comparison of continuous variables between groups, and the chi-squared test or Fisher exact test where appropriate for the comparison of categorized variables. One Way ANOVA and Kruskal Wallis tests were used to compare more than 2 groups. Homogeneity and normality of numeric variables were analyzed using the Kolmogorov-Smirnov test. Data were represented as medians ± standard deviations or binominal percentages where appropriate, if not stated otherwise. Significance was assigned at a p value of less than 0.05.

## RESULTS

### Demographics

A total of 694 patients were entered into the database. The median age at presentation was 54 (16-86) with a female preponderance (n=563; 81.1%). The rate of asymptomatic patients was 25% (n=172). Nearly 21% (n=147) of patients had a history of renal stones, 5.2% (n=36) of patients had a history of fracture, while 2.3% (n=16) were reported to have brown tumors. There were 8 (1.2%) patients with Multiple Endocrine Neoplasia Type I or II, or with a familial-related disease. The median preoperative serum PTH level was 232 (70-8068) pg/mL, while serum total Ca level was 11.4 (7.6-16.2) mg/dL. Vitamin D deficiency was shown in 320 (46.1%) patients.

Characteristics of patients by region are shown in Table 1. There were significant regional differences. The rate of existence of preoperative symptoms was highest in the south-eastern part of Turkey (100%) and lowest in Bulgaria and Eastern Turkey (50%; p<0.001). Patients from Bulgaria and the Turkish Black Sea had the highest median serum Ca levels (11.6 and 11.7 mg/dL, respectively), while South-East Turkey had the lowest (10.9 mg/dL; p<0.001). There was a significantly lower measured p level (2.4 mg/dL) in patients from Eastern Turkey, when compared to other 7 regions (p<0.001).

### Localization procedures

Preoperative localization studies were performed in 660 (95.1%) cases. Ultrasound, sestamibi scintigraphy, CT and MRI were carried out in 89.2%, 86.3%, 39.2%, and 40.2% or 619, 599, 272, and 279 patients, respectively. Apart from each, ultrasound and scintigraphy were positive in 519 (83.8%) and 498 (83.1%) cases, respectively. There were no significant regional differences in these characteristics (Table 1).

### Surgery

Overall, 66% (n=458) of patients underwent a conventional bilateral or unilateral approach. The use of the minimally invasive approaches was low (n=236, 34%) throughout the regions. There was a statistically significant difference between patients from the regions with respect to surgical procedure applied (p<0.001) (Table 1).

### Pathology

Eighty-seven percent of patients (n=606) had an adenoma and 90% (n=542) of these had a single adenoma. Hyperplasia was detected in 79 (11.4%) patients and cancer in 9 (1.3%) patients. The median adenoma size was 16 mm (5-65). There was a statistically significant difference in the median values of adenoma size, having a trend towards larger adenomas in region 4, where the median size was 20 mm (7-60) compared to other regions (p=0.001).

### Complications, persistence or recurrence

Unadjusted postoperative morbidity following surgery and/or rates of recurrence did not differ between regions (Table 1). The rate of persistence was statistically insignificant between regions. Postoperative bleeding occurred in 5 (0.7%) patients. Three (0.4%) patients had postoperative vocal cord palsy. Another 11 (1.5%) patients suffered from hypocalcemia, requiring calcium and vitamin D supplementation. Persistence and recurrence rates were recorded in 23 (3.3%) and 9 (1.3 %) patients, respectively.

## DISCUSSION

In this study, we hypothesized that incidence, morphological, clinical and biochemical changes of pHPT vary between geographical regions. The rationale behind this was constructed due to different reports from different regions of the world for pHPT. These differences might be due to regional factors like genetics, environmental factors and life habits. We found that significant parameters, differing between 8 defined geographical regions, were preoperative symptoms, serum total Ca and p levels, and diseased gland size.

pHPT is one of the most common endocrine diseases, where the clinical presentation has changed dramatically during the last decades (5,6). The literature on pHPT has previously noted that despite similar pathology, pHPT presents with different clinical characteristics in different populations (1). The study by Kirdak et al. (1) compared preoperative clinical and metabolic findings in pHPT patients and adenoma size between age- and sex-matched patients from 2 different continents. It was reported that patients from Turkey had higher serum PTH levels, more severe bone disease and larger adenomas.

Meanwhile, Turkey, geographically situated both in Asia and Europe Balkans, is a large country with an area of 783.562 km2. It borders the Black, Aegean and Mediterranean Seas, and differs widely in geography, including area, population density and the relative contribution of urban and rural areas. For this reason, Turkey has been divided into seven regions, which were originally defined at the First Turkish Geography Congress in 1941. Another country bordering the Black Sea and Turkey is Bulgaria, which is situated in South-East Europe. The land area of Bulgaria, which has its own geographic features, is 110.550 km2, which matches the geographical area of Turkey.

Until recently, geographic variation has been documented in a variety of health situations and their outcomes (7,8,9,10). Our data confirm that geographic disparities exist in clinical and biochemical data in patients with pHPT, which is unique to our study. Previously, geographic disparity had been questioned by de Lucia et al. (10). They reported higher serum Ca levels and lower bone mineral density scores in Italian patients compared to patients from the United States. The data of our study are not surprising based on the knowledge of extraordinary variations in geographic characteristics of the mentioned regions. Although the baseline demographics of patients across regions did not vary widely, the existence of wide regional disparities in pHPT data is striking. We have documented substantial regional differences in the preoperative serum calcium level, with the highest rates observed consistently in the Black Sea region, both in Bulgaria and the Turkish Black Sea region. This finding raises a series of questions that have not been altogether explored; most notably, do the inhabitants of these regions have a main gene polymorphism related to calcium metabolism and therefore experience higher calcium levels more commonly than patients from other regions? Or is there a nutritional impact, since we know that the water of the Black Sea represents special local types with properties that differ from those of sea water in general? (11) It is well known that Black Sea regions receive more rain throughout the year and that desalinated water entry into the Black Sea is much more than that of the Aegean and Mediterranean Seas (12). Another important characteristic of the Black Sea is that it is the largest water mass with continuous haloclines (13). These factors affect calcium concentrations of the water content, since desalinated water contains nearly 20 times more calcium when compared to salty water (12). On the other hand, it might be a matter of debate whether serum calcium levels are affected by geographical variations. There are large volume studies emphasizing increased levels of serum calcium being involved with younger age (14). This relation will be questioned in our future research.

Our study has a number of other salient findings regarding preoperative biochemical changes and sizes of adenomas. Patients from Eastern Turkey were characterized by the lowest value of serum p level. This distinctive feature in patients living in this region have raised questions regarding whether this is related to dietary factors, since Eastern Turkey has no border with the sea and residents consume less seafood than in other regions. Another salient finding is that pHPT patients from South-East Turkey were all symptomatic. This is quite the contrary of what Adami et al. (5) reported in 2002. According to their paper, the introduction of routine biochemical screening resulted in the increased identification of asymptomatic patients. On the other hand, Wermers et al. (15) documented a decline in the incidence of pHPT after making significant changes in clinical practice and discontinuing serum chemistry panels due to regulatory constraints regarding the use of laboratory panels. Nevertheless, the majority of patients with pHPT were found to remain asymptomatic with mild disease, suggesting routine measurements of calcium. The shift between mild and obvious disease might be related to the difference in regional development status as well, as reported by Kirdak et al. (1). It will be important to monitor the regions of our study, since the underlying factors influencing these notable findings remain unclear. Collaborative epidemiologic studies covering these regions are warranted.

Bulgaria and the Mediterranean region had the largest parathyroid gland removed. Even though large parathyroid tumors, larger than 2 cm, are believed to have an increased risk of atypia (2), this could not be confirmed by the current study since only 9 malignant pHPT patients were reported out of 694 pHPT patients with an inconspicuous distribution between regions. Meanwhile, indirectly, our results suggest that clinical and biochemical markers cannot accurately predict adenoma size. Even this has parallelism with the findings of Randhawa et al. (16), so large prospective studies are warranted on this issue. Why adenomas of residents from Bulgaria or the Turkish Mediterranean were larger remains unclear. We know from Cavallaro et al. (17) that growth factors, like insulin-like growth factor 1, basic fibroblastic growth factor, vascular endothelial growth factor, and transforming growth factor beta 1, seem to play an important role in parathyroid adenoma cell proliferation. An understanding of the balance of these growth factors in parathyroid tumors and contributing genetic disparities between different geographic regions may assist in clarifying the etiologic factors which play a role, since geographic variables can contribute to genetic structure through geographic isolation (18). Further prospective studies are warranted to elucidate possible genetic susceptibility.

There are interesting data regarding the demographics of patients who have larger adenomas. Vitamin D deficiency stimulates parathyroid adenoma growth via higher PTH levels and more parathyroid cells are needed to raise the patient’s serum calcium to the level corresponding to the increased set-point that is characteristic of the disease (19). Whether there is a relation between vitamin D levels and adenoma size is worth investigation. Vitamin D deficiency was reported to be as high as 33.4% in Turkey (20). In our study, this was estimated as 46.1%, showing no significant change across regions in prevalence. Meanwhile, it is of utmost importance to take vitamin D status into account to explain the different clinical presentations of pHPT in different geographic regions (21,22). This issue is noteworthy from the clinical point of view as a deficiency of vitamin D may be associated with more severe clinical expression of pHPT, which may cause otherwise mild asymptomatic cases to show clinical manifestations (5). Few studies have assessed vitamin D status in pHPT patients (22,23,24,25,26,27,28,29). Taking into account that these studies have different patient selection criteria and diagnostic cut-off levels for vitamin D deficiency in pHPT, the reported rates of vitamin D deficiency (levels below 50 nmol/L) were within a wide range, from 33% to 90%. In contrast to these studies, the present investigation in the Turkish Asian-Balkan area revealed vitamin D deficiency in 46.1% of 694 pHPT patients. Those that had the lowest rate of vitamin D deficiency (regions 3,4 and 8) also tended to have the lowest serum calcium levels, as expected. Hence, our study group has started a prospective study evaluating the effect of sunshine exposure, clothing and dietary habits, as well as clinical and biological characteristics of pHPT on vitamin D status.

Major shortcomings of the current study come from its retrospective nature and lack of complete data from all regions. Nevertheless, patients were not uniformly distributed throughout the eight regions and the patients were very few in some of them. These are potential confounding factors which are expected from large retrospective studies, but have a negative impact, unfortunately. Secondly, individual center variation may account for some regional variation; a hierarchical model to identify significant underlying center variation as the cause of regional differences is lacking. Thirdly, the present study did not provide any data regarding conversion rates and average follow-up period. Besides, regarding employment for measurement of the main parameters, the study is lacking any cross-calibration between centers. Finally, and maybe most importantly, the variations identified here need to be further investigated to determine whether these changes are attributable to geographic variation by increasing sample size.

In conclusion, our results represented an initial attempt to understand which geographic factors are associated with pHPT and suggested that variations in habitat conditions between geographic regions may affect the clinical and pathological features of pHPT. In addition to raising awareness about the regional disparity of pHPT, our goal with continued research is to characterize further regional differences in pHPT and to determine possible etiologies for these differences. Clinicians should be more vigilant in assessing patients living in the Black Sea region both in Bulgaria and Turkey as well as in South East Turkey for the signs and symptoms of pHPT.

## Figures and Tables

**Table 1:**
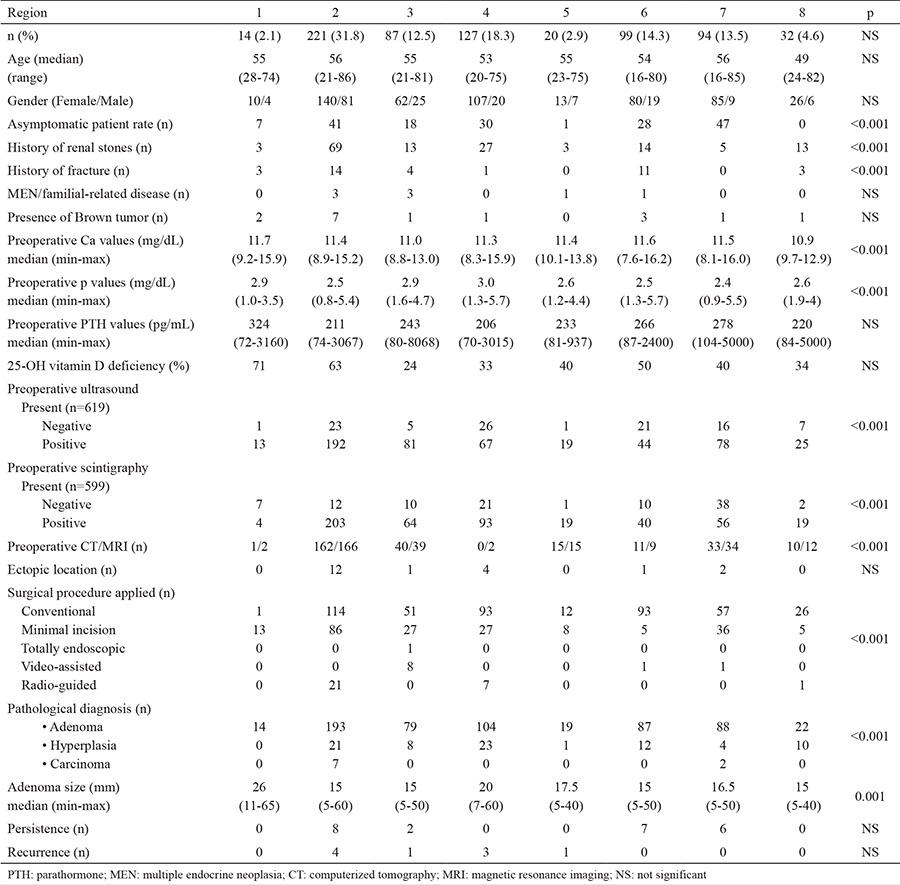
Characteristics of patients by regions
